# Luteolin inhibits diffuse large B-cell lymphoma cell growth through the JAK2/STAT3 signaling pathway

**DOI:** 10.3389/fphar.2025.1545779

**Published:** 2025-02-20

**Authors:** Xin-Zhuo Zhan, Yi-Wen Bo, Yu Zhang, Hai-Dong Zhang, Zhi-Hao Shang, Hui Yu, Xiao-Li Chen, Xiang-Tu Kong, Wan-Zhou Zhao, Timo Teimonen, Tao Liu, Meng-Yi Lu, Ye Yang, Shan-Liang Sun, Hai-Wen Ni

**Affiliations:** ^1^ Department of Hematology, Affiliated Hospital of Nanjing University of Chinese Medicine, Nanjing, China; ^2^ School of Traditional Chinese Medicine, Beijing University of Chinese Medicine, Beijing, China; ^3^ School of Medicine and Holistic Integrative Medicine, Nanjing University of Chinese Medicine, Nanjing, China; ^4^ School of Traditional Chinese Medicine, Nanjing University of Chinese Medicine, Nanjing, China; ^5^ Dongzhimen Hospital, Beijing University of Chinese Medicine, Beijing, China; ^6^ The Nanjing Han & Zaenker Cancer Institute (NHZCI), OG Pharmaceuticals, Nanjing, Jiangsu, China; ^7^ Aqsens Health Oy, Turku, Finland; ^8^ Department of Biostatistics, School of Public Health, Nanjing Medical University, Nanjing, China; ^9^ National and Local Collaborative Engineering Center of Chinese Medicinal Resources Industrialization and Formulae Innovative Medicine, Jiangsu Collaborative Innovation Center of Chinese Medicinal Resources Industrialization, Jiangsu Key Laboratory for High Technology Research of TCM Formulae, Nanjing University of Chinese Medicine, Nanjing, China; ^10^ State Key Laboratory of Natural Medicines, China Pharmaceutical University, Nanjing, China

**Keywords:** luteolin, diffuse large B-cell lymphoma, Janus kinase 2/signal transducer and activator of transcription 3, mechanism, traditional Chinese medcine

## Abstract

Luteolin, a flavonoid present in botanical drugs, plants, and dietary sources, has demonstrated anticancer properties against various tumors, yet its role in diffuse large B-cell lymphoma (DLBCL) remains unclear. This study aimed to uncover the molecular mechanism of luteolin in DLBCL treatment using a combination of *in vitro* and *in vivo* experiments and computational analysis. Human DLBCL cell lines U2932 and OCI-LY10 were utilized to assess luteolin’s impact on cell growth, apoptosis, cell cycle progression, and the modulation of JAK2/STAT3 pathway proteins. *In vivo*, a U2932 tumor-bearing nude mice model was employed to evaluate luteolin’s antitumor efficacy and its effects on JAK2/STAT3 pathway protein expression. Additionally, molecular dynamics simulations were conducted to explore the interaction between luteolin and JAK2. The findings revealed that luteolin significantly suppressed cell proliferation, induced apoptosis, and arrested the cell cycle at the G2/M phase in both cell lines. In the mouse model, luteolin effectively inhibited tumor growth and downregulated the expression of phosphorylated JAK2 and STAT3 without altering the total protein levels of JAK2 and STAT3. Computational analysis indicated stable binding of luteolin to JAK2. Collectively, these results suggest that luteolin’s anti-DLBCL activity may be mediated through the regulation of the JAK2/STAT3 signaling pathway, positioning it as a potential therapeutic agent for DLBCL.

## 1 Introduction

Diffuse Large B-Cell Lymphoma (DLBCL) represents the most prevalent form of aggressive malignant lymphoma globally, constituting approximately 35% of all newly diagnosed malignant lymphoma cases annually (Sehn and Salles, 2021). Over the past decade, first-line R-CHOP (rituximab, cyclophosphamide, doxorubicin, vincristine, and prednisone) chemotherapy regimens, or similar protocols, have markedly enhanced clinical outcomes for patients with DLBCL (Zhang et al., 2023). Nonetheless, the highly heterogeneous nature of DLBCL, coupled with its intricate pathogenesis, results in drug resistance or rapid disease progression in at least 30% of patients, leading to a shortened survival rate (Kim et al., 2023; Younes et al., 2023). Consequently, there is a critical need to identify safe and efficacious alternative therapeutic agents that specifically target the key pathogenic mechanisms of DLBCL.

Luteolin, a flavonoid compound, is naturally occurring in various botanical drugs, including *Prunella vulgaris L.* (commonly known as Selfheal or Xiakucao in traditional Chinese Medicine, TCM, belonging to the Lamiaceae family) and *Scutellaria barbata* D. Don (Ban Zhi Lian, also a member of the Lamiaceae family), as well as in certain dietary sources (Rakoczy et al., 2023). Extensive research has demonstrated that luteolin exhibits potent anticancer properties, particularly against breast, colon, and lung cancer (Zhang and Ma, 2024; Potočnjak et al., 2020; Ahmed et al., 2019). Although less extensively studied in lymphoma, a study by Gharbaran et al. indicated that luteolin can induce cytotoxicity in a mixed cellular classic Hodgkin’s lymphoma model, specifically the KMH2 cell line, through the caspase-mediated apoptotic pathway (Gharbaran et al., 2020). In our previous work, we observed that luteolin, at specific concentrations, effectively inhibits the proliferation of the human DLBCL cell line OCI-LY10 (Zhan et al., 2024a); however, the underlying mechanisms of this effect have not yet been fully elucidated.

Janus kinase (JAK) constitutes a family of intracellular non-receptor tyrosine kinases, comprising four members: JAK1, JAK2, JAK3, and tyrosine kinase 2 (TYK2) (Tian et al., 2024; Roskoski Jr, 2022). The Signal transduction and activator of transcription (STAT) proteins represent a downstream target of the JAK family, consisting of seven primary members: STAT1, STAT2, STAT3, STAT4, STAT5A, STAT5B, and STAT6 (Xin et al., 2020). Among these, STAT3 emerges as a pivotal transcription factor, closely associated with processes of tumorigenesis, metastasis, and drug resistance (Zou et al., 2020). While multiple kinases contribute to the activation of STAT3, JAK2 stands out as a critical upstream molecule that governs STAT3 activation within a variety of tumor cells (Hedvat et al., 2009; Lu et al., 2023). Extensive research has identified small molecule compounds, including the organosilicon compound DCZ0858 and the JAK2 inhibitor TG101209, as agents capable of exerting anti-lymphoma effects by modulating the JAK2/STAT3 signaling pathway (Xu et al., 2022; Zhang et al., 2021a; Lu et al., 2020). Our research team has demonstrated that the innovative patent formula Qiling Baitouweng Tang, along with its active monomers, pulsatilla saponin A and quercetin, can influence the proliferation of DLBCL cells and induce apoptosis by regulating the JAK2/STAT3 pathway, underscoring the intimate relationship between the inflammatory pathway, JAK2/STAT3, and DLBCL (Zhan et al., 2024b; Liu et al., 2023a; Liu et al., 2023b).

**FIGURE 1:**
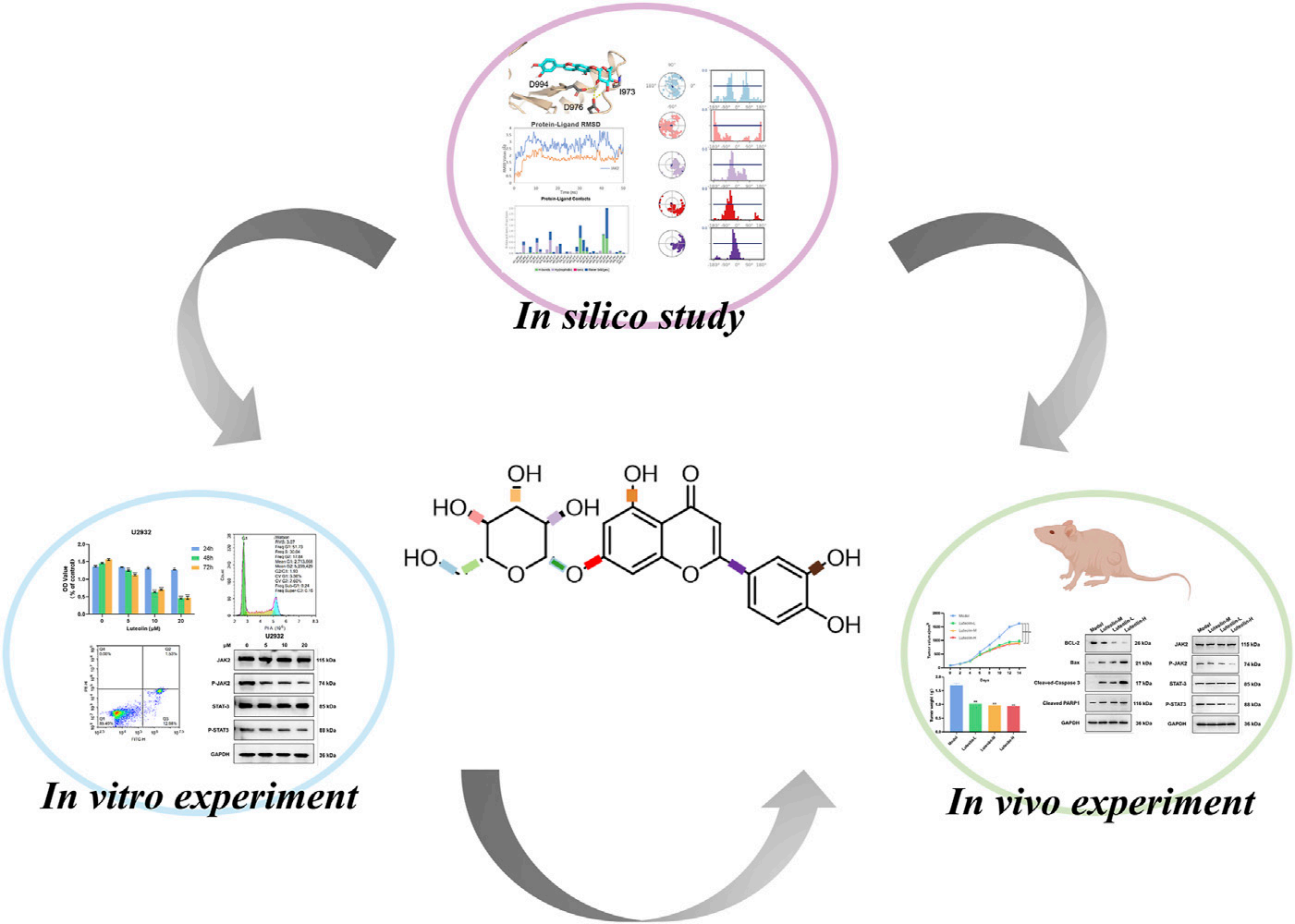
The flow chart of this study.

The objective of this study was to elucidate the potential mechanisms by which luteolin exerts its anti-DLBCL effects through the JAK2/STAT3 signaling pathway, utilizing a combination of *in vitro* and *in vivo* assays, as well as computational studies. The research aimed to establish a theoretical foundation for the clinical development of single or small molecule inhibitors derived from TCM (Figure 1).

## 2 Materials and methods

### 2.1 Reagents

Luteolin was purchased from Saen Chemical Technology Co., Ltd (Shanghai, China). Roswell Park Memorial Institute (RPMI) 1640 medium (Lot#8120384) and fetal bovine serum (FBS) (Lot#2152441P) were purchased from Gibco, US. Dimethyl sulfoxide (DMSO) was purchased from Nanjing Liangwei Biotechnology Co. (Nanjing, China); Annexin V-FITC/PI Cell Apoptosis Kit (No. BA11100), Cell Cycle Analysis Kit (No. BA40100) were purchased from Nanjing Enjing Biotech (Nanjing, China). The BCA protein concentration determination kit (No. P10010) was purchased from Shanghai Beyotime Biotech Inc (Shanghai, China). The EnoGeneCell™ Counting Kit-8 (CCK-8) (No. E1CK-000208-100), radioimmunoprecipitation assay (RIPA) cracking liquid (Lot#20220209), antibodies for Bcl-2 (No. E2511313), Bax (No. E2515633), Cleaved-Caspase 3 (No. E11-0104L), Cleaved PARP (Cleaved-Asp214) (No. E11-0365L), JAK2 (No. E2511497), JAK2 (Phospho-Tyr570) (No. E11-0499A), STAT3 (No. E2510955), STAT3 (Phospho-Tyr705) (No. E2614980) GAPDH (No. E20-53445) were purchased from EnoGene (Nanjing, China). The sodium chloride injection (Lot#L122091103) was purchased from Sichuan Kelun Pharmaceutical Co., Ltd.

### 2.2 Instruments

HERAcell 150i CO_2_ incubator (Thermo, the US); DMi1-1 inverted microscope (Leica, Germany); 3K15 low-temperature high-speed centrifuge (Sigma, the US); Infinite 200 pro automatic microplate reader (Tecan, Switzerland); PowerPac Basic electrophoresis apparatus, Mini-PROTEAN Tetra electrophoresis tank (Bio-Rad, the US); Tanon 5200 Multi automatic chemiluminescence/fluorescence image analysis system (Nanjing MGD Biotechnology Co., Ltd.); Gallios flow cytometer (Beckman, the US).

### 2.3 Cell culture

Human DLBCL cell lines (U2932 and OCI-LY10) were presented by Prof. Li’s laboratory from the Department of Hematology at the First Affiliated Hospital of Nanjing Medical University. U2932 and OCI-LY10 cells were cultured in RPMI-1640 complete medium containing 10% fetal bovine serum and 1% penicillin-streptomycin in an incubator at 37°C with 5% CO_2_ and humidity.

### 2.4 CCK-8 assay

The effect of luteolin on the proliferation of U2932 cells was examined by dilution of luteolin at gradient concentrations (0–100 μM). U2932 cells were seeded in 96-well plates at a density of 1.5 × 10^6^/mL, and the medium was changed every 24 h. CCK-8 assay was performed 72 h later. 10 μL CCK-8 solution was added to each well and continued to culture for 2 h. Then the absorbance (OD) at a wavelength of 450 nm was measured by a microplate reader, and the cell proliferation inhibition rate and IC_50_ value were calculated to determine the grouping of subsequent *in vitro* experiments. Then, the absorbance of luteolin on U2932 and OCI-LY10 cells at three time points of 24 h, 48 h and 72 h was detected.

### 2.5 Annexin V-FITC/PI double-staining assay

U2932 and OCI-LY10 cells treated with luteolin for 24 h were collected. After centrifugation at 1000 rpm for 5 min (140 mm radius), the supernatant was discarded, the cells were resuspended in PBS, and the above steps were repeated. Then 1 × 10^6^/mL suspension was prepared by adding 500 μL 1 × Annexin V Binding Solution. 5 μL Annexin V-fluorescein isothiocyanate (FITC) was added and stained with 5 μL propidium iodide (PI) solution for 15 min. The apoptosis of each group was analyzed by flow cytometry.

### 2.6 Cell western immunoblot assay

The treated cells were washed twice with PBS, resuspended with an appropriate amount of RIPA lysate, and lysed for 30 min at 4 °C. After the protein concentration was determined using the BCA protein concentration determination kit according to the manufacturer’s instructions, the appropriate volume was added with loading buffer at a 1:5 ratio and denatured in a boiling water bath at 100 °C for 10 min. The target proteins were separated by polyacrylamide gel electrophoresis and then transferred to the membrane for antibody incubation. The dilution ratio of primary antibody was 1:1000, and the dilution ratio of secondary antibody was 1:5000. The density of each band was quantified using ImageJ software, and the GAPDH band was used as an internal control.

### 2.7 Flow cytometry analysis

Cells were added and collected as in item 2.5 and washed by adding 1 mL of precooled PBS. The cells were then fixed in 70% ethanol for 24 h at 4°C. The cells were centrifuged at 1000 r·min^-1^ for 10 min and the supernatant was discarded. The samples were then washed with PBS, and 0.5 μL of PI was added to each tube for staining. The cells were incubated at room temperature in the dark for 30 min, and the cell cycle of each group was detected by flow cytometry within 24 h.

### 2.8 Animal experiment

Thirty-five SPF female BALB/c nude mice (18–20 g, 4–6 weeks of age) were purchased from the Comparative Medical Center of Yangzhou University. The nude mice were housed in the SPF laboratory animal room for 1 week. A total of 35 BALB/c nude mice were subcutaneously inoculated with 0.1 mL U2932 cell suspension (5 × 10^6^ cells/mL) in the right axilla. The groups were grouped according to the previous concentrations of luteolin in animal experiments in the field of anti-tumor, as well as our previous pre-experiment (Wu et al., 2023). When the tumor volume of nude mice reached 80 mm^3^, the nude mice were randomly divided into 4 groups (n = 5): model group (0.2 mL normal saline per mouse), luteolin low, medium and high dose groups (12.5 mg/kg, 25 mg/kg and 50 mg/kg) were given 0.2 mL/20 g body weitht by intraperitoneal injection twice a day for 14 days.

The general health status of nude mice was observed every day during administration. The long and short diameters (mm) of U2932 xenograft tumors were measured by vernier caliper every other day, the tumor volume (mm^3^) was calculated, and the changes in body weight (g) of nude mice were recorded. After 14 days of drug administration, the nude mice were euthanized, the tumor tissues were stripped, weighed, and the tumor inhibition rate (%) was calculated. Tumor inhibition rate (%) = (tumor weight of model group-tumor weight of drug administration group)/tumor weight of model group ×100%. The tumor volume (TV = 1/2×a × b^2^) and relative tumor volume (RTV = V_t_/V_0_) were calculated by measuring the long and short diameters of the dissected tumors. According to the RTV results, the relative tumor proliferation rate (T/C (%) = T_RTV_/C_RTV_×100%) was calculated to evaluate the antitumor activity of luteolin in tumor-bearing nude mice. The animal experimental protocol was conducted by the Ethics Committee of the Nanjing Han and Zaenker Cancer Institute (Nanjing, China) under the certificate number OGKQSPF/SQ-96.

### 2.9 Tissue western blotting assay

Tumor tissue sections with a volume of about 10 mm^3^ were taken from each group, and an appropriate amount of ice-soluble RIPA lysate was added to extract total protein. The tissue was then ground on a grinder three times for 1 min each. Protein concentration was determined using the BCA protein concentration determination kit. Western blot was used to detect apoptosis and protein expression of JAK2/STAT3 signaling pathway, as described in Section 2.6.

### 2.10 Molecular modeling simulation

All molecular docking simulations were performed using Glide (version 78,011, Schrödinger, Inc., New York, NY, 2013). For each ligand, up to 30 poses were generated using the Glide XP mode. The docking simulations were carried out with prepared ligands and predefined grids, as defined by the co-crystalized ligand. The docking score for each ligand was calculated as the sum of the Glide Gscore and a state penalty, which accounts for the protonation or tautomeric state of the ligand. Additionally, the Emodel scoring function was employed to further evaluate the plausibility of different protein‒ligand docking poses, providing complementary insights into the docking results.

### 2.11 Molecular dynamics simulation

The Desmond protocol module in Schrodinger employed the OPLS3 force field for Molecular Dynamics (MD) simulations, utilizing the SPC solvent model (Harder et al., 2016). To maintain clarity among solvent molecules, an orthorhombic periodic boundary condition was implemented with minimized box volume (Figure 8B) (Nam, 2014). Subsequently, the system was reevaluated and neutralized by the addition of Na + ions. Sampling was carried out at a temperature of 300 K and constant pressure consumption, with samples taken every 250 picoseconds (ps) over a total simulation time of 50 nanoseconds (ns) resulting in 200 samples for further systematic analysis. The remaining settings remained at their default values, and the quality of the MD simulation was assessed using Simulation Interaction Diagram tools.

### 2.12 Statistical analysis

The experimental results were expressed in the form of mean ± standard deviation (SD) and statistically analyzed by GraphPad Prism 8.0 software. Each group of experiments was repeated three or more times. The *t*-test was used to compare the differences between the two groups. One-Way ANOVA was used to test quantitative data between different groups. *P* < 0.05 indicated statistical difference.

## 3 Results

### 3.1 Luteolin inhibited the proliferation of human DLBCL cells

**FIGURE 2:**
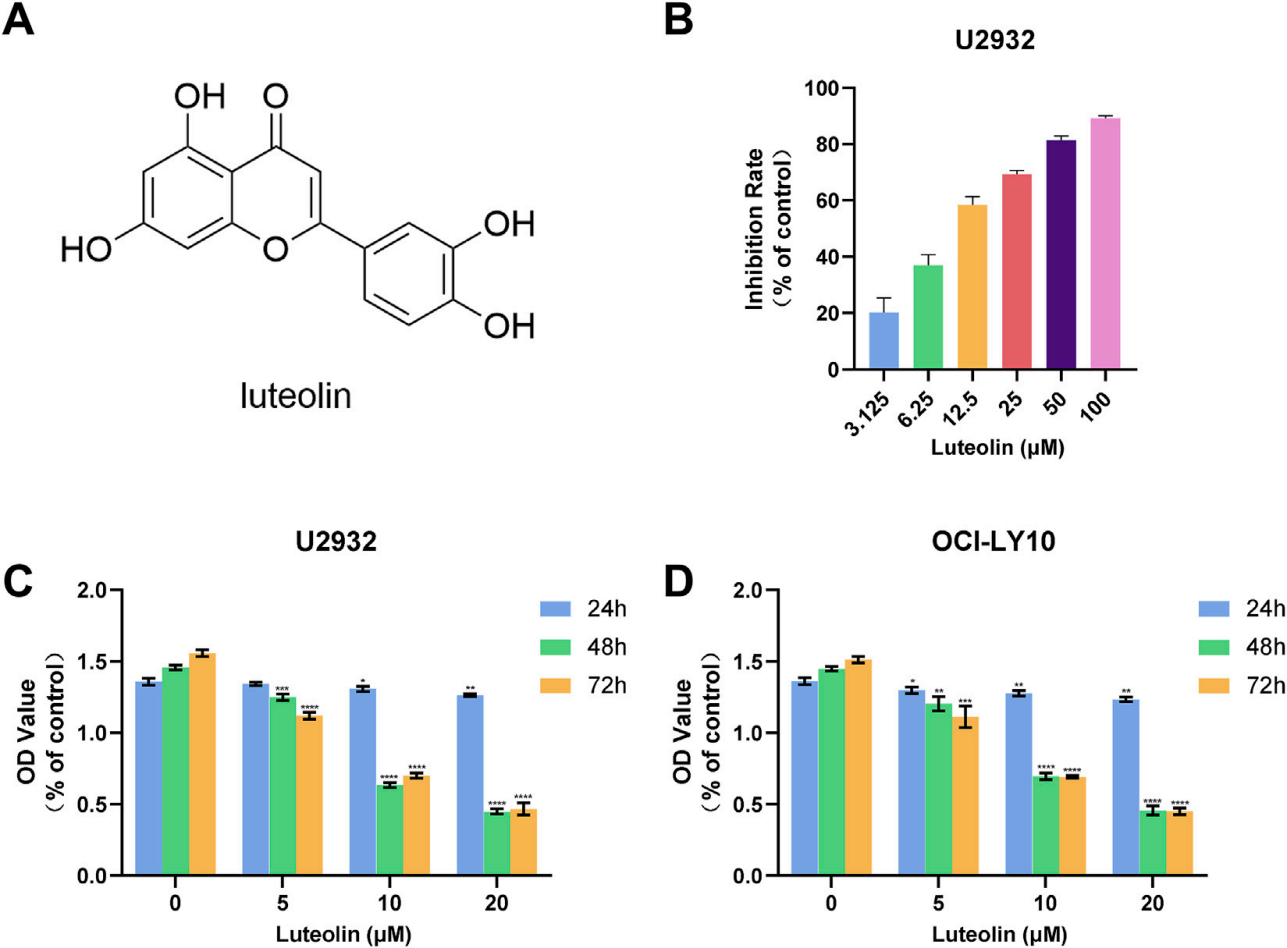
Luteolin inhibited the growth of DLBCL cells. **(A)** Chemical formula of luteolin **(B)** Effect of luteolin (0–100 μM) on the proliferation of U2932 cells after 72 h **(C, D)** Effects of luteolin (0, 5, 10 and 20 μM) on the growth of U2932 **(C)** and OCI-LY10 **(D)** DLBCL cells at different time points (24, 48 and 72 h) (x ± s, n = 3; ^*^ *P* < 0.05, ^**^ *P* < 0.01, ^***^ *P* < 0.001 and ^****^ *P* < 0.0001 vs. the blank control group).

In our investigation into the anti-proliferative effects of luteolin (Figure 2A) on human DLBCL cells, we utilized the CCK-8 assay to assess cell viability. Specifically, we treated U2932 cells with luteolin for 72 h and determined that it significantly inhibited cell proliferation, with an IC_50_ value of 10.91 μM, compared to the control group (Figure 2B). Consistent with our previous findings, luteolin also markedly reduced the viability of OCI-LY10 cells, with an IC_50_ value of 12.03 μM (Zhan et al., 2024a). Based on these IC_50_ values, we proceeded with *in vitro* experiments using luteolin concentrations of 0, 5, 10, and 20 μM.

To further elucidate the inhibitory effects of luteolin on DLBCL cell proliferation, we exposed both U2932 and OCI-LY10 cell lines to luteolin at concentrations of 0, 5, 10, and 20 μM over a period of 24, 48, and 72 h. The CCK-8 assay was employed to monitor cell proliferation, and the data revealed that luteolin dose-dependently and time-dependently suppressed the growth of both U2932 and OCI-LY10 cells (Figures 2C, D). These results underscore the potential of luteolin as a therapeutic agent against DLBCL, highlighting its ability to impede cell proliferation in a manner that is both concentration- and time-dependent.

### 3.2 Luteolin induced apoptosis of human DLBCL cells

**FIGURE 3:**
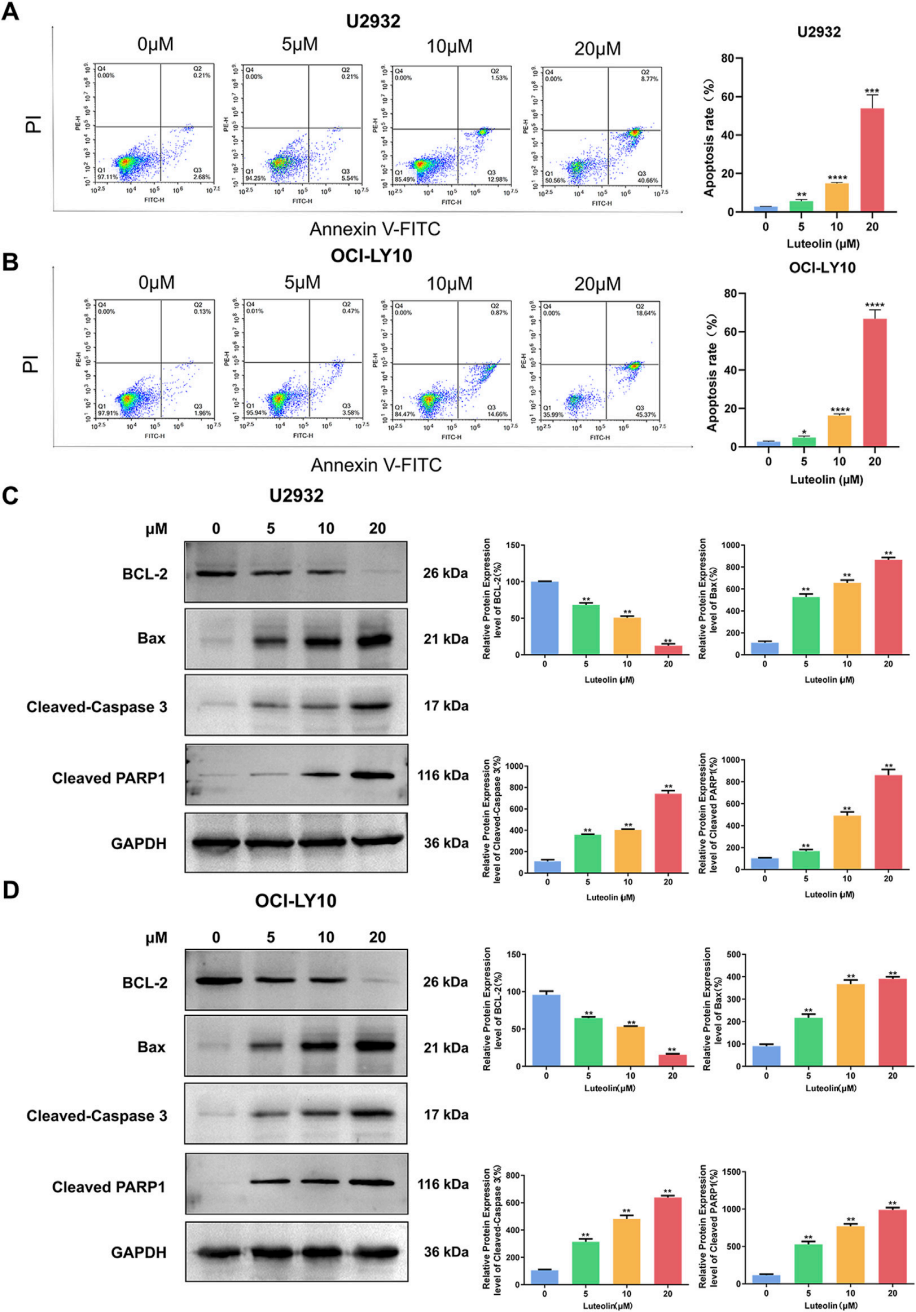
Luteolin induced apoptosis in DLBCL cells. **(A, B)** Luteolin (0, 5, 10 and 20 μM) induced apoptosis in U2932 **(A)** and OCI-LY10 **(B)** cells. **(C, D)** Effect of luteolin (0, 5, 10 and 20 μM) on the levels of anti-apoptotic protein BCL-2 and pro-apoptotic proteins Bax, Cleaved-Caspase 3 and Cleaved PARP1 in U2932 **(C)** and OCI-LY10 **(D)** cells. The difference was statistically significant (x ± s, n = 3; ^*^ *P* < 0.05, ^**^ *P* < 0.01, ^***^ *P* < 0.001 and ^****^ *P* < 0.0001 vs. the blank control group).

To assess the apoptotic effects of luteolin on human DLBCL cell lines U2932 and OCI-LY10, we employed Annexin V-FITC/PI double staining and observed a significant dose-dependent increase in apoptotic cell numbers following a 24-h treatment with luteolin at concentrations of 5, 10, and 20 μM, as compared to the untreated control group. Specifically, the apoptosis rates for U2932 cells were calculated as (5.59 ± 0.88)%, (14.91 ± 0.39)%, and (53.82 ± 7.11)%, respectively (Figure 3A). Similarly, the apoptosis rates for OCI-LY10 cells were (4.85 ± 0.78)%, (16.39 ± 0.79)%, and (66.74 ± 4.71)%, respectively (Figure 3B).

To elucidate the molecular mechanisms underlying luteolin-induced apoptosis in DLBCL cells, we analyzed the expression of key apoptosis-related proteins using Western blot. After a 24-h exposure to luteolin at the same concentrations, we observed a decrease in the expression of the anti-apoptotic protein BCL-2 and an increase in the expression of the pro-apoptotic protein Bax in a dose-dependent manner when compared to the blank control group. Additionally, the protein levels of cleaved PARP and Cleaved-Caspase 3 were found to be elevated in the luteolin-treated groups (Figures 3C, D). These findings suggest that luteolin induces apoptosis in DLBCL cells by modulating the expression of proteins involved in the intrinsic apoptotic pathway, thereby promoting cell death.

### 3.3 Luteolin induced cell cycle arrest in human DLBCL cells

**FIGURE 4:**
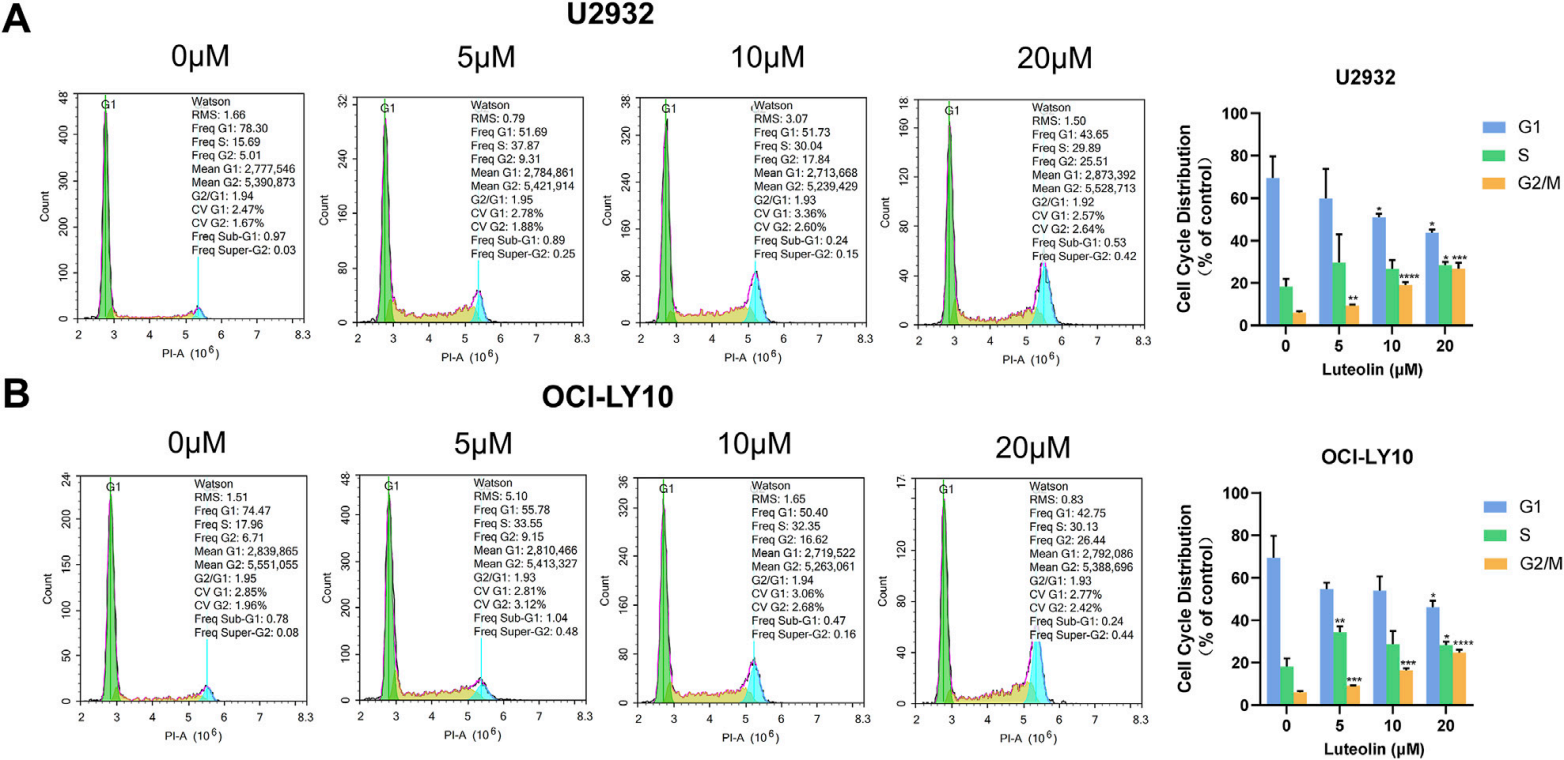
Luteolin induced G2/M phase arrest in DLBCL cells. Cell cycle distribution of human DLBCL cells U2932 **(A)** and OCI-LY10 **(B)** treated with luteolin (0, 5, 10 and 20 μM). The difference was statistically significant (x ± s, n = 3; ^*^ *P* < 0.05, ^**^ *P* < 0.01, ^***^ *P* < 0.001 and ^****^ *P* < 0.0001 vs. the blank control group).

Utilizing flow cytometry, we analyzed the cell cycle distribution of U2932 and OCI-LY10 cells following a 24-h treatment with luteolin at concentrations of 5, 10, and 20 μM. The results indicated a significant increase in the proportion of cells arrested in the G2/M phase in a dose-dependent manner (Figure 4). Specifically, the percentages of U2932 cells in the G2/M phase after treatment with 5, 10, and 20 μM luteolin were (9.39 ± 0.52)%, (19.15 ± 1.32)%, and (26.87 ± 2.69)%, respectively. Similarly, the percentages of OCI-LY10 cells arrested in the G2/M phase were (10.01 ± 0.71)%, (19.00 ± 0.64)%, and (26.67 ± 1.28)%, respectively, when exposed to the same concentrations of luteolin.

These data demonstrate that luteolin induces a dose-dependent G2/M phase arrest in both U2932 and OCI-LY10 cells, suggesting that the compound’s anti-proliferative effects may be mediated, at least in part, through the disruption of normal cell cycle progression at the G2/M transition. This cell cycle perturbation could be a critical mechanism by which luteolin exerts its antitumor activity against DLBCL.

### 3.4 The anti-DLBCL effect of luteolin was related to JAK2/STAT3 signaling pathway

**FIGURE 5:**
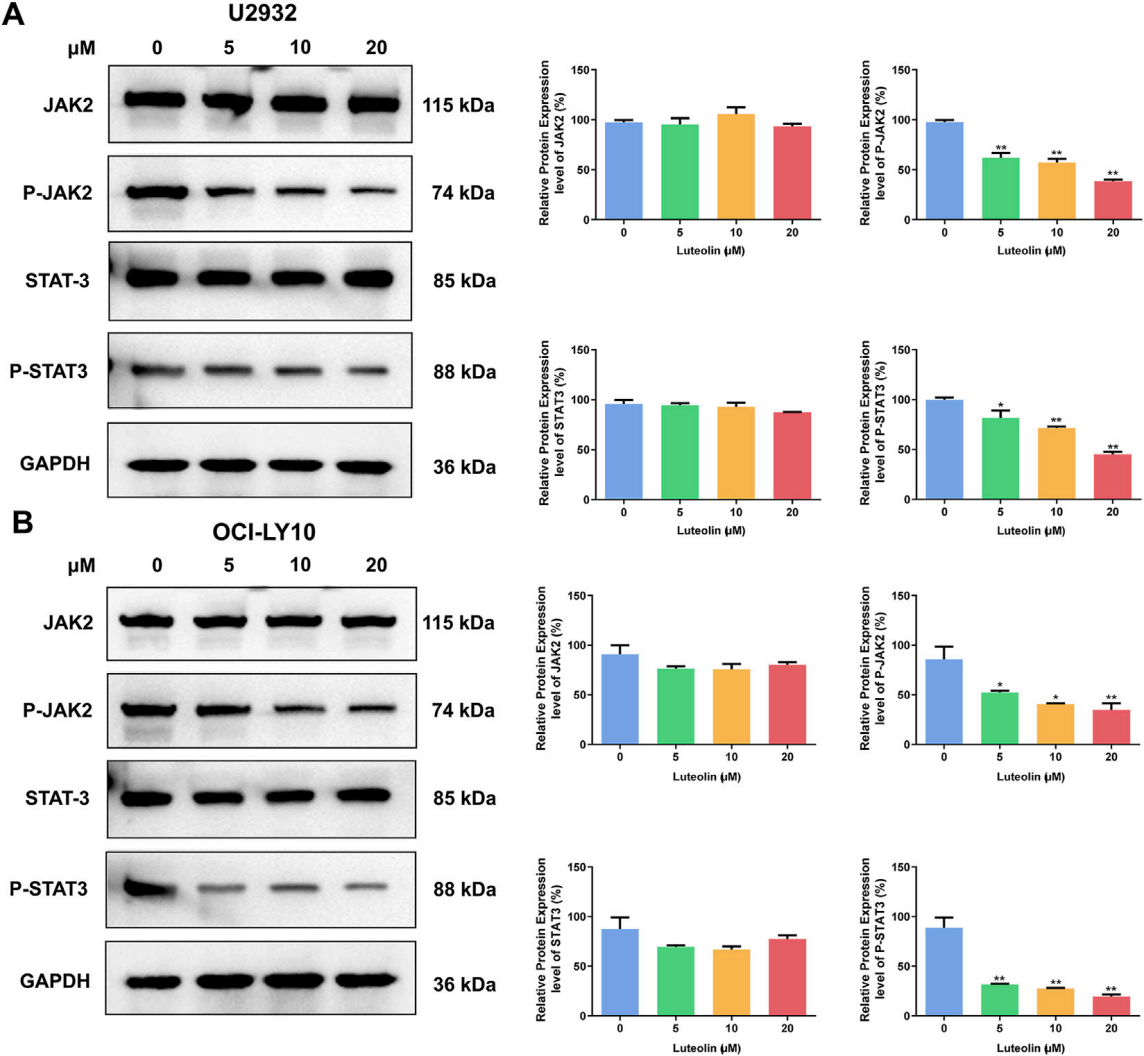
Luteolin inhibited the phosphorylation of JAK2/STAT3. Effect of luteolin (0, 5, 10 and 20 μM) treatment on the expression of JAK2/STAT3 pathway-related proteins in human DLBCL cells U2932 **(A)** and OCI-LY10 **(B)**. The difference was statistically significant (x ± s, n = 3; ^*^ *P* < 0.05 and ^**^ *P* < 0.01 vs. the blank control group).

Upon exposure to luteolin for 24 h at concentrations of 5, 10, and 20 μM, U2932 and OCI-LY10 cells exhibited a dose-dependent decrease in the expression levels of phosphorylated JAK2 (p-JAK2) and phosphorylated STAT3 (p-STAT3) proteins, as determined by Western blot analysis (Figure 5). In contrast, the total protein levels of JAK2 and STAT3 remained unchanged in the presence of luteolin, indicating that the compound specifically targets the activation state of these signaling molecules rather than their overall expression. This observation suggests that luteolin may modulate the JAK2/STAT3 signaling pathway by inhibiting the phosphorylation of JAK2 and STAT3, which could be a key mechanism underlying its antitumor effects in DLBCL. The preservation of total JAK2 and STAT3 protein levels implies that luteolin’s action is selective, potentially leading to fewer off-target effects and highlighting the pathway’s importance in the context of DLBCL cell signaling.

### 3.5 Luteolin inhibited U2932 tumor growth in nude mice by regulating JAK2/STAT3 signaling pathway

**FIGURE 6:**
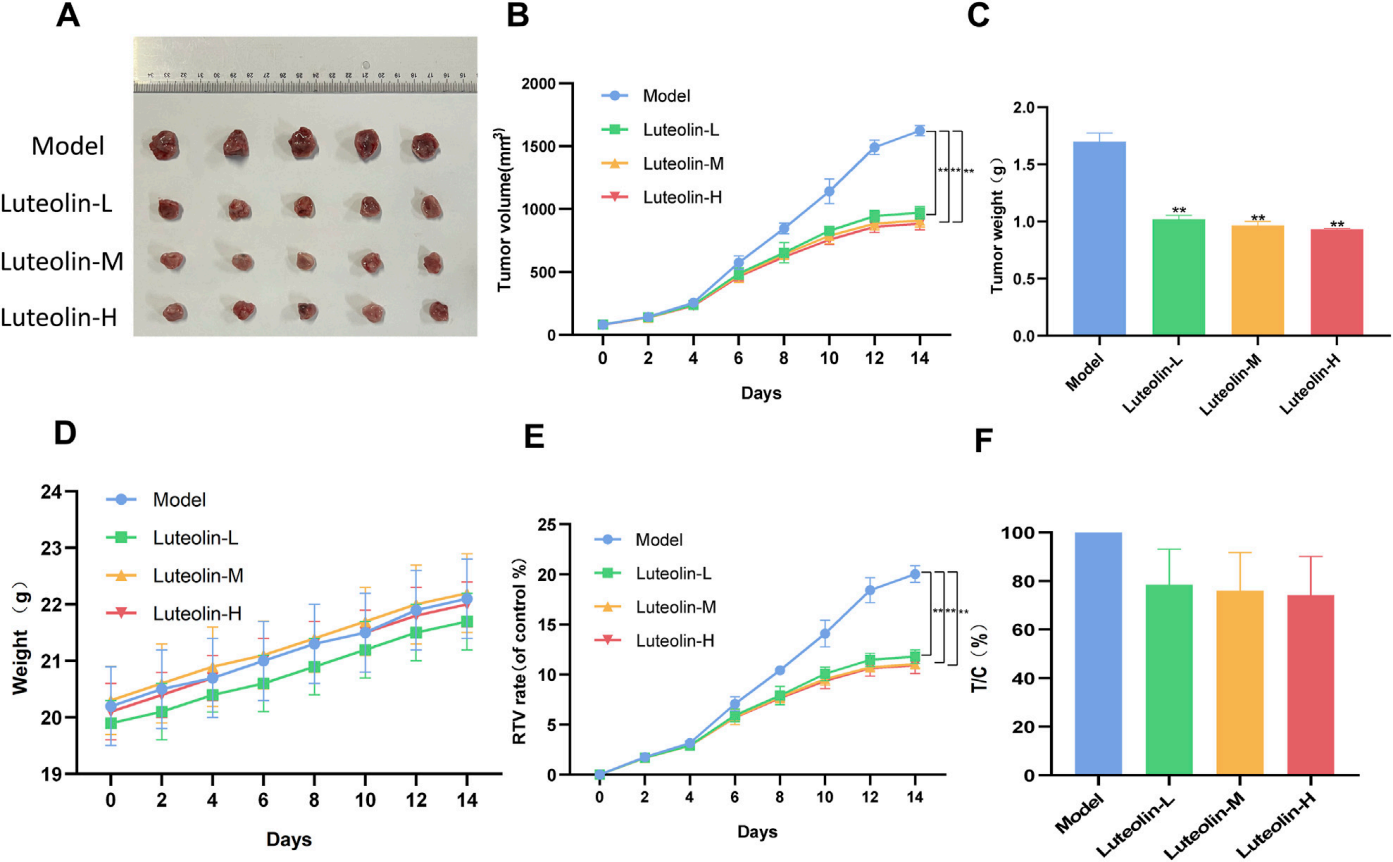
Luteolin (12.5 mg/kg, 25 mg/kg and 50 mg/kg) inhibited tumor growth in U2932 transplanted nude mice. **(A)** Photographs of tumor of mice in each group 14 days after drug administration. **(B)** Tumor growth curve. **(C)** Tumor weight. **(D)** Body weight curves of mice. **(E)** Relative tumor volume (RTV) **(F)** Relative tumor proliferation rate (T/C) (n = 5/group; *P* < 0.05 vs. the model group).

*In vivo* antitumor efficacy of luteolin was assessed using a U2932 tumor-bearing nude mice model. Luteolin was administered at doses of 12.5 mg/kg, 25 mg/kg, and 50 mg/kg, and its effects were compared against a control model group. The treatment resulted in a significant dose-dependent reduction in both tumor volume and tumor weight, indicating a potent inhibitory effect on tumor growth (Figures 6B–F). Notably, luteolin had minimal impact on the body weight of the nude mice, suggesting that the treatment was well-tolerated and did not lead to significant systemic toxicity.

After a 14-day treatment period, the nude mice were euthanized, and the tumors from each treatment group were harvested for further protein expression analysis (Figure 6A). This procedure allowed for the examination of the molecular effects of luteolin directly within the tumor microenvironment, providing valuable insights into its mechanism of action *in vivo*. The preservation of body weight and the observed reduction in tumor burden underscore the potential therapeutic index of luteolin as an anticancer agent in the context of DLBCL.

**FIGURE 7:**
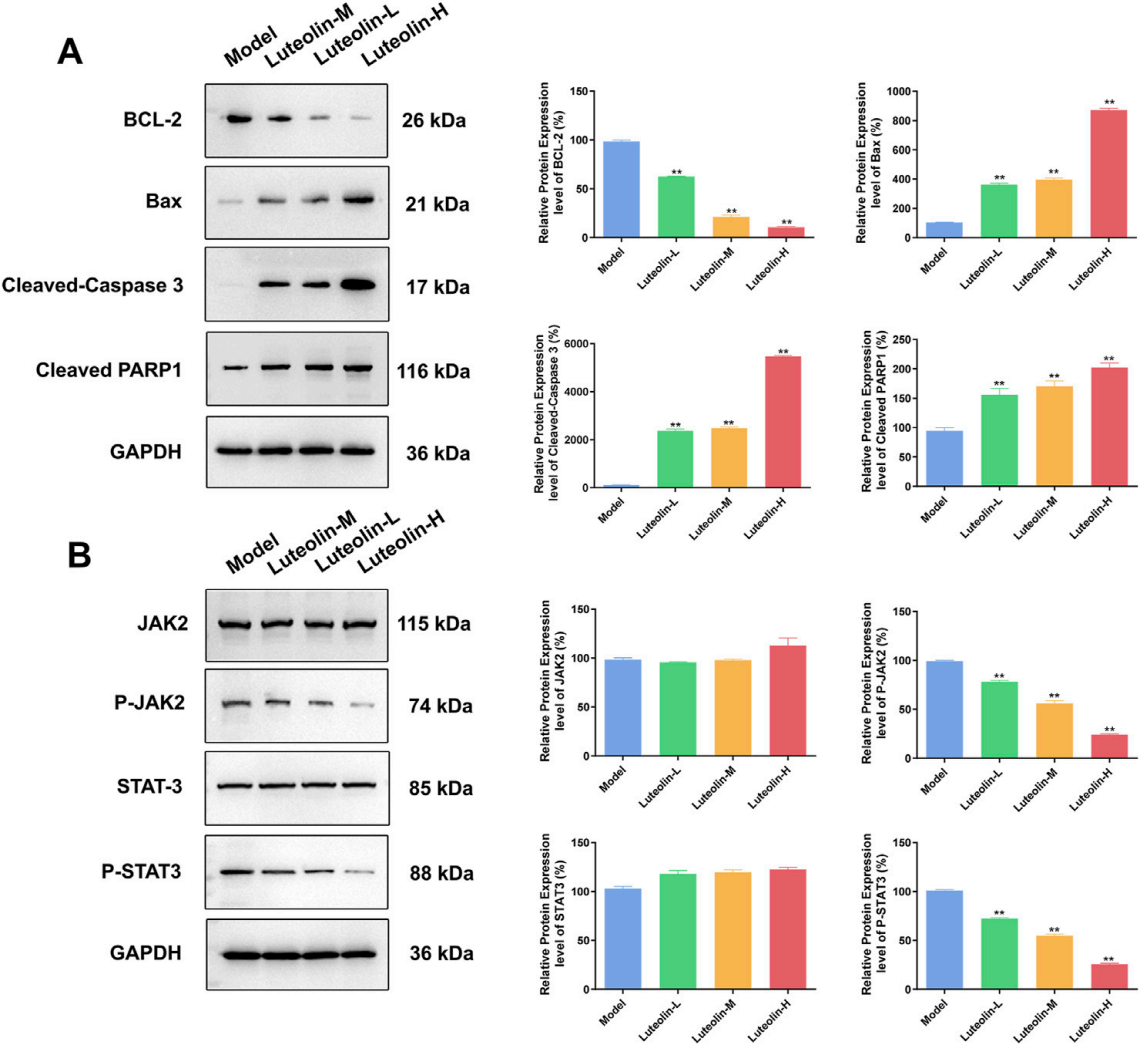
Effect of luteolin (12.5 mg/kg, 25 mg/kg and 50 mg/kg) on U2932 tumor-burdened nude mice in the tumor apoptosis related proteins (BCL-2, Bax, Cleaved-Caspase 3 and Cleaved PARP1) **(A)** and the effect of JAK2/STAT3 pathway-related proteins (JAK2, P-JAK2, STAT3 and P-STAT3) expression **(B)** The difference was statistically significant (x ± s, n = 3; ^*^ *P* < 0.05 and ^**^ *P* < 0.01 vs. the blank control group).

Western blot analysis was conducted to evaluate the expression levels of apoptosis-related proteins and proteins within the JAK2/STAT3 signaling pathway in tumor tissues harvested from U2932 tumor-bearing nude mice following a 14-day treatment with luteolin at doses of 12.5 mg/kg, 25 mg/kg, and 50 mg/kg. The findings revealed that, in comparison to the model group, the luteolin-treated groups exhibited a significant dose-dependent decrease in the expression of the anti-apoptotic protein BCL-2 and the phosphorylated forms of JAK2 (p-JAK2) and STAT3 (p-STAT3). Concurrently, there was a significant dose-dependent increase in the expression of pro-apoptotic proteins Bax, as well as the activation of caspase-dependent apoptotic pathways, as evidenced by the increased levels of Cleaved-Caspase 3 and Cleaved PARP1 (Figure 7). Notably, the total protein levels of JAK2 and STAT3 remained unchanged, indicating that luteolin specifically modulates the activation state of these proteins rather than their absolute expression levels. These results suggest that luteolin’s antitumor effects *in vivo* are associated with the induction of apoptosis and the inhibition of the JAK2/STAT3 signaling pathway, which are key events in the regulation of cell survival and proliferation. The dose-dependent nature of these changes further supports the potential therapeutic utility of luteolin in modulating critical cellular processes in DLBCL.

### 3.6 Molecular dynamics simulation of luteolin binding to JAK2

**FIGURE 8:**
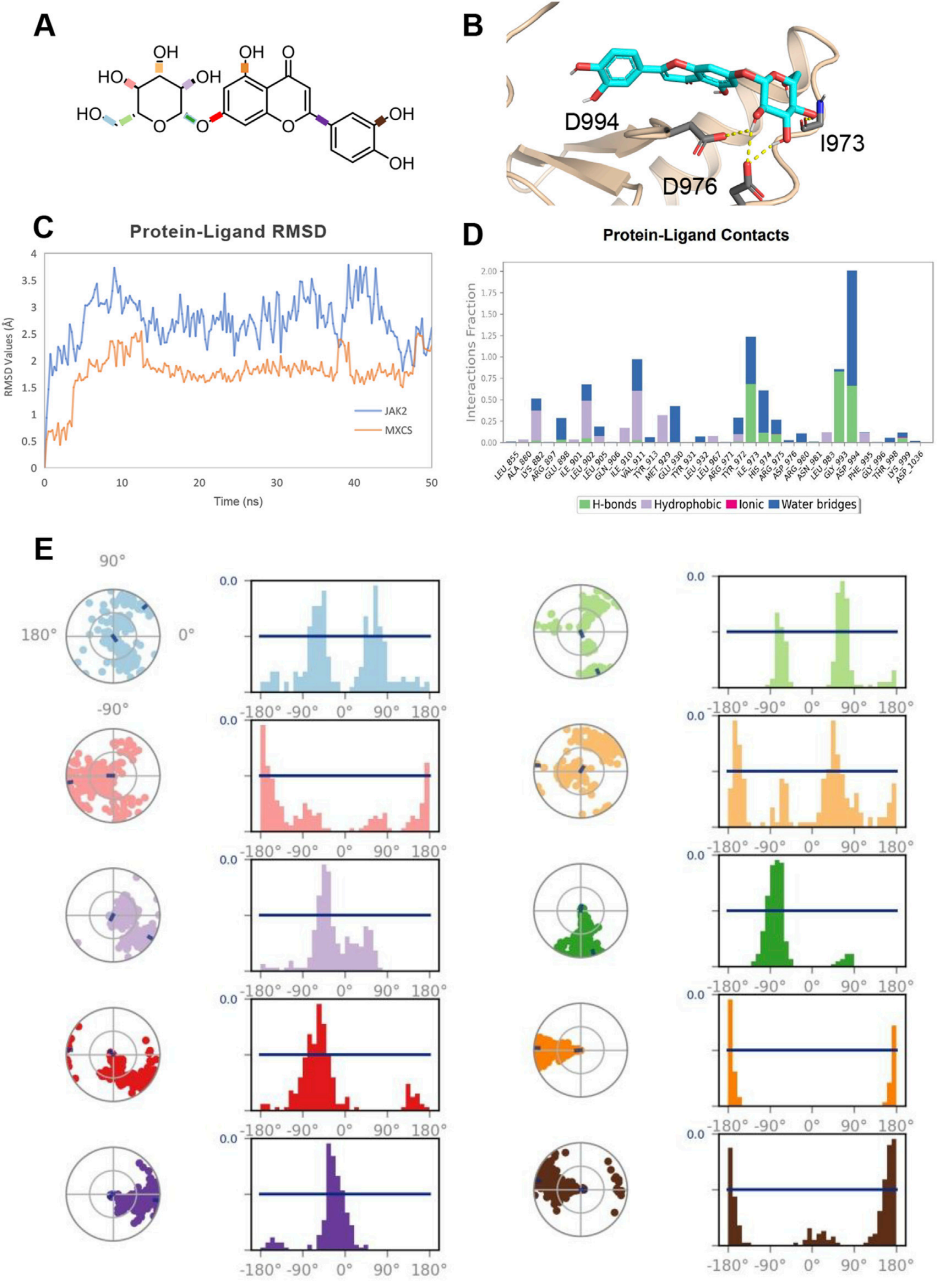
**(A)** 2D Chemical Structure of luteolin. The molecular structure of the luteolin compound is illustrated, highlighting its key chemical groups and rotatable bonds. **(B)** Detailed Binding Mode of luteolin with JAK2. Luteolin is depicted as cyan sticks, and the JAK2 protein backbone is shown in wheat cartoon. Key interacting residues are highlighted as gray sticks, with the corresponding interactions represented by yellow dashed lines. **(C)** Protein-Ligand RMSD Plot. The RMSD (Root-Mean-Square Deviation) values for the JAK2 protein backbone (colored in blue) and luteolin (colored in orange) are tracked over a 50 ns molecular dynamics simulation. The RMSD provides insights into the structural stability and flexibility of the complex throughout the simulation. **(D)** Protein-Ligand Interactions Fractions in MD Simulation. A bar plot visualizing the interaction types (hydrogen bonds, hydrophobic interactions, ionic bonds, and water bridges) between JAK2 and luteolin throughout the 50 ns trajectory. Each interaction type is indicated by a specific color: green for hydrogen bonds, purple for hydrophobic, magenta for ionic, and blue for water bridges. **(E)** Rotatable Bond Analysis of luteolin. Torsion plots showing the conformational evolution of each rotatable bond within luteolin. Each bond is represented by both a dial plot and a corresponding histogram, revealing the flexibility and dynamic behavior of the compound during the simulation.

The molecular dynamics simulation (Figure 8A) of luteolin binding in JAK2 protein (Figure 8B) over 50 ns provides valuable insights into the stability, binding interactions, and conformational flexibility of the compound. The RMSD analysis (Figure 8C) reveals that the luteolin-JAK2 complex both maintained stability throughout the simulation, without obvious fluctuations indicating dynamic conformation change in the binding site. The luteolin ligand shows relatively low RMSD values compared to the JAK2 backbone, suggesting that the ligand remains tightly bound and undergoes limited displacement within the binding pocket. The interaction analysis (Figure 8D) demonstrates that luteolin forms a variety of persistent contacts with JAK2 residues. Hydrogen bonds dominate the interaction landscape, contributing significantly to the compound’s binding stability. Hydrophobic interactions further enhance the ligand’s fit within the active site, while occasional ionic and water-bridge interactions add additional stabilizing factors. Notably, hydrogen bond interactions with key residues such as I973 and D994 play a crucial role in forming these interactions, maintaining more than 30% occupancy throughout the simulation. The rotatable bond analysis (Figure 8E) shows that the conformational freedom of luteolin is reasonably limited within the binding pocket. Most rotatable bonds exhibit distinct conformational preferences, as seen from the narrow peaks in the histogram plots, indicating restricted torsional movements. However, certain bonds retain moderate flexibility, allowing the compound to adapt to subtle structural changes in the binding pocket during the simulation. This balance between flexibility and rigidity contributes to both the specificity and stability of luteolin as a JAK2 ligand. Overall, the simulation suggests that luteolin is a promising ligand for JAK2, demonstrating stable binding, key residue interactions, and controlled flexibility. These results provide a strong foundation for further optimization of luteolin to enhance selectivity and efficacy in targeting JAK2.

## 4 Discussion and conclusion

DLBCL is the most prevalent form of non-Hodgkin lymphoma globally, characterized by its heterogeneity and aggressive nature, with a rising incidence rate (Chapuy et al., 2018). Clinically, DLBCL is typically manifested by the enlargement of lymph nodes, potentially accompanied by symptoms such as unexplained fever, night sweats, and pruritus, necessitating prompt medical intervention (Martelli et al., 2013). In recent years, the incorporation of molecularly targeted therapies, including bortezomib, ibrutinib, and lenalidomide, into the standard R-CHOP (rituximab, cyclophosphamide, doxorubicin, vincristine, and prednisone) regimen has led to some improvement in the prognosis for patients with DLBCL (Denker et al., 2022; Villacampa et al., 2021). Despite these advancements, the management of DLBCL in clinical practice is still confronted with challenges posed by the side effects of these novel agents, such as increased susceptibility to infections, thrombotic events, and cardiotoxicity.

Contemporary TCM employs a variety of therapeutic agents, including compound formulations, single botanical drugs, and small molecule drugs, to combat malignant tumors. These agents are known to potentiate the efficacy of chemotherapeutic regimens and mitigate the associated toxicities (Jung and Cheon, 2024; Zhang et al., 2021b; Ling et al., 2014). Within this context, flavonoids, plant-derived compounds such as quercetin, luteolin, apigenin, and kaempferol, are recognized for their potential anticancer properties (Gupta et al., 2023). Luteolin, in particular, has been demonstrated in previous studies to exert anticancer effects through the inhibition of cell proliferation, suppression of angiogenesis, and retardation of extracellular matrix degradation (Wu et al., 2021; Feng et al., 2020; Fang et al., 2018). Our own prior research has indicated that luteolin significantly inhibits the proliferation of OCI-LY10 cells *in vitro* (Zhan et al., 2024b). Building upon these findings, the present study selected luteolin for further investigation into its molecular mechanisms of action against DLBCL. We observed that luteolin induced apoptosis, arrested the cell cycle at the G2/M phase, and upregulated the expression of pro-apoptotic proteins Bax, Cleaved-Caspase 3, and Cleaved PARP1 in a concentration-dependent manner in both U2932 and OCI-LY10 cells. Conversely, the expression of the anti-apoptotic protein BCL-2 was downregulated, further highlighting the compound’s potential as a therapeutic agent in the treatment of DLBCL.

The JAK2/STAT3 signaling pathway is frequently dysregulated in various cancers and plays a significant role in the progression of malignant tumors (Mengie et al., 2022). Our study specifically targeted the protein markers of the JAK2/STAT3 signaling pathway. Western blot analysis revealed that luteolin significantly downregulated the expression of p-JAK2 and p-STAT3 in a concentration-dependent manner, while the protein expression levels of total JAK2 and STAT3 remained largely unchanged. *In vivo* experiments conducted in nude mice bearing U2932 tumors demonstrated that luteolin inhibited tumor growth and had a minimal effect on body weight, with the inhibitory effect becoming more pronounced as the dose increased. Western blot was employed to assess the expression of Bax, Cleaved-Caspase 3, and Cleaved PARP1 in tumor tissues treated with luteolin. The expression levels of BCL-2, p-JAK2, and p-STAT3 decreased with increasing doses, while the protein expression of JAK2 and STAT3 did not significantly change. The increasing focus on small molecule drugs targeting JAK2 and STAT3 is evident in current research (Zhuang et al., 2021; He et al., 2017; Lee et al., 2015). Luteolin has been shown to bind to STAT3 with stability (Li et al., 2023). Molecular dynamics simulations were used to evaluate the stability of the luteolin-JAK2 complex, and the results indicated that luteolin binds to JAK2 in a stable conformation. Therefore, our data confirm that luteolin may inhibit the proliferation of DLBCL cells, induce apoptosis, and suppress tumor growth through the modulation of the JAK2/STAT3 signaling pathway.

In conclusion, our study has, for the first time, identified that luteolin exerts an anti-DLBCL effect through the modulation of the JAK2/STAT3 signaling pathway. This discovery lays a theoretical foundation for considering luteolin as a potential anticancer agent for the treatment of DLBCL. However, it is important to note that our research did not investigate the synergistic inhibitory effects of luteolin in combination with other anticancer agents on DLBCL. This represents a limitation of our study and an area that warrants further exploration in future research. The potential for combination therapies could enhance the efficacy of luteolin and provide additional treatment options for DLBCL patients.

## Data Availability

The original contributions presented in the study are included in the article/supplementary material, further inquiries can be directed to the corresponding authors.

## Abbreviations

DLBCL, Diffuse large B-cell lymphoma; JAK2, Janus kinase 2; MD, Molecular Dynamics; R-CHOP, Rituximab, cyclophosphamide, doxorubicin, vincristine, and prednisone; RMSD, Root-Mean-Square Deviation; RPMI, Roswell Park Memorial InstituteRTV, Relative tumor volume; SD, Standard deviation; STAT3, Signal transducer and activator of transcription 3; TCM, Traditional Chinese medicine; TV, Tumor volume; TYK2, Tyrosine kinase 2.
